# ‘I Think it Just Made Everything Very Much More Intense’: A Qualitative Secondary Analysis Exploring The Role Of Friends and Family Providing Support to Survivors of Domestic Abuse During The COVID-19 Pandemic

**DOI:** 10.1007/s10896-021-00292-3

**Published:** 2021-06-28

**Authors:** Alison Gregory, Emma Williamson

**Affiliations:** 1 Centre for Academic Primary Care, Bristol Medical School, University of Bristol, Canynge Hall, 39 Whatley Rd, Clifton, Bristol, BS8 2PS UK; 2 Centre for Gender and Violence Research, School for Policy Studies, University of Bristol, Bristol, UK

**Keywords:** Domestic Abuse, Informal Support, Friends and Family, Bystanders, Pandemic

## Abstract

The COVID-19 pandemic, and associated social restrictions, have amplified women’s experiences of domestic abuse (DA). In usual times, female DA survivors reach out to those around them (friends, family members, neighbors, and colleagues) for support. Accessing of both professional and informal support by survivors has increased during the pandemic. Informal supporters are often deeply invested and immersed in situations of DA because of the closeness of relationships. The accounts of informal supporters are rarely sought, yet these are people who may have a considerable awareness of what is happening. The aim of this study was to explore how the pandemic had impacted people’s assessment of abusive situations and their ability to provide informal support. This paper reports a secondary analysis of qualitative data collected in 2020 in England. The data were gathered in 18 in-depth interviews with people who knew a female friend, relative, neighbor, or colleague who had experienced DA. The age range of participants was 25–69 years, three were men and fifteen were women. A reflexive thematic analysis was carried out. Findings indicated: (i) the pandemic had changed people’s ability to read situations and assess risk (ii) perpetrators were exploiting the pandemic to further abuse (iii) within the context of the pandemic there was additional challenge to offering support (iv) informal supporters found creative ways to remain in-touch and to continue offering support. Further research with informal supporters is needed to ascertain how best to support and equip people, without imposing an impossible burden.

## Introduction

When the COVID-19 pandemic hit the UK, and announcements were made about social distancing restrictions, lockdowns, and closures of places of education and work, domestic abuse (DA) specialist organisations were justifiably apprehensive. Newspaper stories from China (Feng, 2020; Wanqing, 2020) indicated that fears around rising numbers of DA incidents, escalation of violence and abuse, and increasing demand for support were being realised. These patterns were soon replicated globally, with world-wide newspaper reports and subsequent research indicating similar pictures (Every-Palmer et al., 2020; Graham-Harrison et al., 2020; Hamadani et al., 2020; Mohler et al., 2020; Office for National Statistics, 2020; Sánchez et al., 2020; Sediri et al., 2020; Taub, 2020; The Economist, 2020). What was less clear in the media was that spikes in DA related to the amplification of abuse for existent survivors, rather than more people suddenly becoming abusive (Williamson et al., 2020).

In England, Women’s Aid, a national charity working to end domestic abuse against women and children^1^ described the unfolding situation as ‘a perfect storm’; not because COVID-19 was causing DA, but because many elements of the pandemic, including measures designed to protect people from the virus, had worsened and intensified abuse, and had closed down routes to support and safety (Birchall et al., 2021; Davidge, 2020). Indeed, there has been emerging evidence globally that all forms of abuse experienced by survivors prior to the pandemic have increased in frequency and severity. This includes physical, emotional, psychological, verbal, and sexual violence and abuse, financial/economic abuse, control and restriction of freedom, cyber abuse, stalking, surveillance and monitoring behaviours (Bracewell et al., 2020; Hamadani et al., 2020; Jung et al., 2020; Moreira & Pinto da Costab, 2020; Sediri et al., 2020). Moreover, novel, pandemic-specific forms of abuse have been employed by perpetrators, such as refusing to take precautions or threatening to intentionally expose survivors and their children to the virus; coughing or spitting on survivors; forcing survivors to follow strict isolation rules; threatening to throw survivors out of the house if they have symptoms; using lockdown measures to prevent survivors from leaving, or to force their way back into survivors’ homes; and blocking access to children (Davidge, 2020; Gingrich, 2020; Newberry & Santa Cruz, 2020). There are also indications that more women in the UK have been killed by partners, ex-partners, and adult family members over this period (Ingala Smith, 2020; Office for National Statistics, 2020). Additionally, children living in DA households have faced greater suffering; witnessing and directly experiencing more abuse (Davidge, 2020). In light of this woeful picture, how have services been coping with demand?

Unfortunately, the pandemic struck at a time when specialist DA services in the UK had been substantially cut, or underfunded, for many years. A decade of austerity measures not only reduced direct funding for specialist DA organisations, but also increased demand on the voluntary sector as a whole, by reducing statutory service provision (Davidge, 2019; Reis, 2018). This resulted in insufficient provision for DA survivors and children; reducing spaces for people to seek refuge and safety, and limiting community-based support (Miles & Smith, 2018). Moreover, specialist DA services for marginalised communities are in a particularly precarious position. These services have been most impacted by austerity cuts (Thiara & Roy, 2020), and the communities they serve have been greatly affected by the pandemic (Birchall et al., 2021). Imkaan, a UK-based organisation dedicated to addressing violence against Black and minoritized women and girls, highlights the disproportionate effects of the dual pandemics (COVID-19 alongside violence against women and girls) on marginalised communities:

*…structural inequality reproduces disproportionately across diverse communities and exacerbates existing racialised inequalities. For any woman and girl with protected characteristics, the two pandemics increase her risks at multiple interlocking levels (*Imkaan, 2020*)*

As the reality of the pandemic dawned, national specialist domestic and sexual violence and abuse organisations in the UK collectively raised concerns about the impending rise in demand, and requested funding to address this (Statement on Covid-19, 2020). In late Spring 2020, the UK government invested substantial financial support in the sector, but there has been uncertainty about how sustained this support will be (Davidge, 2020). In addition to specialist services’ capacity to cope with demand, there have also been concerns raised that some survivors may be unable to seek support from professional organisations at this time (Office for National Statistics, 2020), for reasons such as increased time spent with and surveillance by perpetrators of abuse (Davidge, 2020; Sánchez et al., 2020).

Formal support provision is, of course, only part of the picture. Prior to the pandemic, research indicated that women experiencing abuse tended to reach out to those around them (their friends, family members, neighbors, and colleagues) considerably more than to professionals (Ansara & Hindin, 2010; Parker & Lee, 2002). Additionally, the rates of disclosure and help-seeking increase as abuse escalates (Fanslow & Robinson, 2010; Sylaska & Edwards, 2014). Reaching out for informal support may feel like a smaller step to take, appear less daunting, or be easier to access than professional help (Gregory, 2014). Research has shown that, when this informal support is perceived as positive, in addition to improving safety, health, wellbeing, and quality of life, it can also lead to increased help-seeking from professionals (Coker et al., 2002, 2003; Goodman et al., 2005; Plazaola-Castano et al., 2008). Thus, informal support is an incredibly important part of the picture. Moreover, research has shown that during the pandemic, people experiencing abuse have spoken with friends and family members more than they did before (Iob et al., 2020). It is perhaps unsurprising then, that during the pandemic when less-usual, more creative solutions and responses are being considered, that the significance of community responding has come to the fore. UK initiatives, such as, *Ask me*^2^ community ambassador training run by Women’s Aid have garnered attention during this period, and the government launched a campaign (#YouAreNotAlone) to encourage communities to demonstrate solidarity with those experiencing DA (UK Home Office, 2020). Likewise, specialist DA services have produced additional resources^3^ for members of the public and have broadcast messages on social media urging people to notice situations of abuse happening around them (for example, #ReachIn). Moreover, academic papers published on the topic of DA since COVID-19 are increasingly recommending (though sometimes in rather cursory ways) community-based initiatives. In particular, encouraging people to check on those around them, and to take action, as a partial solution in the current crisis (Sánchez et al., 2020; Telles et al., 2020; van Gelder et al., 2020).

Friends, family members, neighbors, and colleagues may be well-placed to help. Several of the primary advantages of informal support relate to access: (i) permission and ease of access—a survivor can contact someone close to them at almost any time and, because it is an established relationship, it may be more comfortable than reaching out to a professional (ii) legitimacy of contact – it may be safer for survivors to maintain a connection with someone providing informal support than a professional, not least because they have other, legitimate reasons for being in touch (iii) proximity – informal supporters often live in the locality of survivors, particularly neighbors and friends. They may witness some of what is happening first-hand and have greater opportunities for creatively engineering face-to-face interactions. Thus, informal supporters are potentially valuable assets; offering help which may be vital and, being advantageously situated. However, given the substantial training expected of professionals who encounter DA, is it reasonable to expect members of the public to simply respond and get it right? Our previous research on this topic, has shown that friends, relatives, neighbors, and colleagues find DA situations complex and confusing, and that their physical and mental health, relationships, and safety may be affected by their involvement (Gregory, 2014; Gregory et al., 2017a; Gregory et al., 2017b; Gregory, 2017c; Gregory et al., 2019). Additionally, the person they are supporting is someone they are emotionally invested in, and the support period may be lengthy. This can challenge people’s understanding of their role within the situation and the response needed from them (Latta, 2008; McKenzie et al., 2020).

The focus of this research is intentionally on support for *women* who have experienced DA. This is both due to the gender asymmetry regarding the prevalence and impact of DA (Ansara & Hindin, 2010; Office for National Statistics, 2016), and because the role of informal supporters is much more apparent in the lives of female survivors (Ansara & Hindin, 2010; Hester et al., 2012). Additionally, it is important to acknowledge, that the burden of providing informal support falls largely to women. Our previous research has shown that women supporters (especially friends and sisters of survivors) knew considerably more about DA situations than male counterparts (Gregory et al., 2019). Similarly, other researchers have highlighted that survivors disclose and seek support almost exclusively from female friends, mothers, and sisters. (Rose et al., 2000; Sylaska & Edwards, 2014). These findings, prior to COVID-19, are noteworthy because during the pandemic women have, willingly or otherwise, picked up a larger share of caring roles, and will undoubtedly be impacted long-term by this additional caregiving (The Fawcett Society, 2020; The Feminist Alliance for Rights, 2020). Thus, we have a situation during the pandemic, where women are experiencing escalating violence and abuse, and are more likely to seek support from people informally, but where the people from whom they are seeking support, predominantly other women, are already stretched by increases in more general caring responsibilities.

Informal supporter perspectives are seldom sought regarding DA situations. By understanding the experiences of informal supporters, we can work towards empowering and equipping them, providing a double gain; they themselves are supported, so they cope better, and they can thus provide better help to survivors. As an additional benefit, since direct research with survivors has been limited during the pandemic for safety reasons, informal supporters can provide side-line accounts of what has been happening.

The aim of the research was to foreground narratives from people providing informal support to DA survivors during the pandemic, with the following research questions: (i) How has the pandemic changed the ways that informal supporters are able to offer support, (ii) How has the pandemic changed situations of domestic abuse.

## Methods

A secondary analysis of qualitative data collected in 2020 during the COVID-19 pandemic is reported in this paper. The original study was an investigation exploring the needs of friends, family members, neighbors, and colleagues of women experiencing DA, in order for them to feel equipped and supported to provide optimal informal help. Since the study was underway when the pandemic hit, we included additional questions to explore how the pandemic had impacted people’s assessment of the abusive situation and their ability to provide support. For this study, the UK Home Office definition of domestic abuse was used:

*Any incident or pattern of incidents of controlling, coercive or threatening behaviour, violence or abuse between those aged 16 or over who are or have been intimate partners or family members regardless of gender or sexuality. This can encompass, but is not limited to, the following types of abuse: psychological, physical, sexual, financial & emotional* (UK Home Office, 2013)

### Recruitment and Data Collection

The original study recruited participants primarily from the Southwest of England, but in later stages was extended to include people from across England. This widening of participation was a result of the shift to online methods of recruitment, and to remote participation, as a consequence of lockdown and social distancing measures. Participants were initially recruited using poster advertisements in local healthcare and community venues (including community centres, foodbanks, and libraries), and latterly through social media, email, and e-newsletter publicity by the research team, collaborating partners, and relevant third sector organisations. This publicity highlighted the study’s webpage, which provided potential participants with the lead researcher’s (AG) contact details. Potential participants were screened to assess eligibility, and also any risk of harm which might result from their participation. The eligibility criteria for this study were: (i) participants were aged 16 years or above, and (ii) that they knew a woman (a friend, family member, neighbor, or colleague) who had experienced DA according to the UK Home Office definition.

For the original study, focus groups were the intended mode of data collection, but this evolved to remote individual interviews as COVID-19 restrictions were introduced. Since the focus groups took place prior to lockdown and social distancing measures, only interview data is reported in this secondary analysis. A topic guide was used in semi-structured interviews with participants, and once the UK social restriction measures came into effect (mid-March 2020), an additional question was included to enquire about the impact and effects of the pandemic upon the DA situation: ‘How has the situation changed during the pandemic? (both the survivor’s situation and how the participant has been able to offer support)’. For the original study, 34 participants were recruited, 10 contributing to focus group discussions, and 24 taking part in individual interviews with the first author (AG). Interview participants were given the choice to use Skype (with or without the camera setting) or to be interviewed over the telephone. The interviews were audio-recorded (using an external recorder rather than in-software recording), transcribed *verbatim* and imported into NVivo12 software.

In the interviews, many people highlighted the impacts of the pandemic on the DA situation faced. This subset of interviews ranged in length from 34 to 105 min, and while some participants spoke peripherally about the pandemic and related constraints, for others it was the predominant focus. It would be usual to conduct a secondary analysis on the data provided in these interviews after the primary analysis had been completed. However, given the relevancy and timeliness of this piece of work, we decided to conduct the secondary analysis ahead of the overall study, the findings from which will be published in due course.

The original study was granted ethical approval by the Research Ethics Committee in the School for Policy Studies at the University of Bristol in October 2019. Subsequent amendments were submitted, in order that the study could: (i) include individual interviews for data collection (ii) recruit using additional online means, and (iii) include pandemic-specific questioning. These amendments were approved in January 2020 and July 2020. Permission had already been granted for remote interviewing at participants’ request.

### Data Analysis

Secondary analyses of qualitative data are used by researchers to answer new or additional research questions from those intended in the original study (Heaton, 2008). The analyses are considered ‘an efficient alternative’ to collecting more data from the same or different participants; a way of capitalising on an existing data set, particularly if the population studied are seldom heard (Chew-Graham et al., 2012; Long-Sutehall et al., 2010; Tate & Happ, 2018). Moreover, since further data collection is unnecessary, potential burden from research participation, particularly around difficult and sensitive topics, is avoided (Chew-Graham et al., 2012; Long-Sutehall et al., 2010). The secondary analysis reported in this paper explored how the pandemic, and related restrictions, had impacted on situations of DA from the perspectives of those providing informal support (friends, family members, neighbors, and colleagues). Additionally, the analysis explored the effect on informal supporters’ ability to offer help and to cope themselves. A thematic analysis (TA) was undertaken (Braun & Clarke, 2006); an approach which has been used to good effect for secondary study of primary research relating to DA (D'Amore et al., 2021; Gregory et al., 2020; Hashimoto et al., 2021). More specifically, since the epistemology of the research was informed by critical realism, a perspective which indicates that multiple ‘truths’ can exist and be known, a reflexive form of TA was employed (Braun & Clarke, 2020; King & Horrocks, 2010). In line with Braun and Clarke’s approach, our methods were thus ‘*creative, reflexive and subjective with researcher subjectivity understood as a resource, rather than a potential threat to knowledge production’* (Braun & Clarke, 2019).

The first author (AG) identified transcripts which included accounts relating to the COVID-19 pandemic. Relevant data were extracted and coded independently by both authors, and through thoughtful discussion, the final themes were agreed, honed, and elaborated. In the presentation of themes, quotes from participants are included; the parentheses contain the participant's pseudonym and their relationship to the DA survivor/victim.

## Researcher Reflexivity

Part of ensuring the quality and transparency of qualitative research is for investigators to recognise their subjectivity; the traditions, values, beliefs, and personal qualities they bring to their research. It is also important for readers to have an understanding of the people who conducted the research. To this end: both the authors are white women in their 40s, who have worked in the field of gender-based violence for a combined period of 40 years. The authors employ feminist ideals to underpin their research. Both have previously worked and/or volunteered in the DA specialist sector, and both have provided informal support to survivors.

## Findings

Of the 24 participants interviewed for the original study, 18 spoke about how the pandemic and associated restrictions had impacted on a current DA situation, the aftermath of a DA situation, or their abilities to offer support or to cope with the situation they faced. The people who took part in this subset of interviews were aged 25–69 years, three were men and fifteen were women. Eight of the participants indicated that they themselves had had experiences of domestic abuse in adulthood or childhood. Their relationships with a survivor were: friend (10), neighbour (1), mother (3), sister (1), daughter (1), aunt (1), and cousin (1). For eight of the participants, this was the first situation of domestic abuse they had encountered. In all but one situation, the survivor was female, and the perpetrator of abuse was male. In two cases, the perpetrator was an adult family member—son (1), and mother (1)—and in the remainder, the perpetrator was a partner or ex-partner.

The following themes and subthemes were developed from people’s accounts:

### Reading the Situation and Risk-Assessment

It was clear that many informal supporters felt less sure about what was happening within situations of DA after the pandemic started. It was not that they felt they had previously known everything, but that the pandemic had highlighted specific areas of unsurety or had created a greater overall uncertainty. In many cases, participants appeared unclear about whether the abuse was continuing, whether the forms or patterns of abuse had altered or escalated, and whether the survivor had been communicating all that was happening:

*…the contact has been more sporadic, and more sort of general (Fabian, friend to a survivor)*

*I think it’s the same, except that she’s not seeing her family, even as much as she was before, so I guess that’s… I don’t really know. I haven’t been myself [to see her] for a few weeks, so I’m not sure (Bibi, friend to a survivor of family abuse)*

*I don’t know if the abuse is carrying on or not… she’s the one who is saying that he is not hitting her anymore, but the kids are saying that he’s hitting them, they’re all in their 20s (Ojana, cousin to a survivor)*

This was not the case in all types of relationship; neighbors, in particular, were in positions where they might know considerably more because of the pandemic. With people spending greater amounts of time at home, both through homeworking and spending less social time elsewhere, neighbors were very aware of what was going on next door:

*…we are now all here, closed up, and I just feel it is escalating, it’s becoming more frequent that they are shouting and I’m having to call the police (Maria, neighbor and friend to a survivor)*

One of the most important things about accurately ascertaining what is happening, is an assessment of the level of danger. Participants indicated that they had little information to go on in order to evaluate the risk, and were quite worried by this:

*I know her phone is monitored by her partner. She has to be much more restrained in what she says…I am sure it would be the same for other people in abusive relationships. Their lifelines are probably quite tenuous at the moment. I can imagine an awful lot of conversations, text messages, emails and that kind of thing are closely guarded by abusive partners. That ability to be open is quite affected, which is a worry (Martha, friend to a survivor)*

*…since the lockdown, it’s been difficult to get a feeling of what’s been going on…I thought this can go one of two ways. Either things will calm down because he has her at home. She has to do a bit of work from home, and that’s exactly where he wants her, and she can’t go out, so there isn’t that kind of friction of him saying, ‘you can’t go out’. My actual gut feeling was, ‘I bet he’s loving this’…I do have to sort of press her to say, ‘I’m finding this really hard being locked down’ but whether that’s just generally, anything else is going on in the background, I don’t know. It’s difficult to say, she plays her cards really close to her chest sometimes. The worse things get, the tighter the cards are to her chest. (Fabian, friend to a survivor)*

Instead, people were having to fill in the gaps, regarding both the situation and the impact, using their prior knowledge of the situation, assumptions about factors which may increase or decrease safety, and trying to spot any visible clues:

*I imagine COVID has made things worse ‘cos they’re all in the house together…I think, during COVID, because nobody was working at all and…if they’re not working, they’re at home (Ojana, cousin to a survivor)*

*…at the start of lockdown, I was terrified really that something was going to happen because the three of them, like I say, there’s a lot of friction anyway, and I just thought, ‘what can you do in that situation if they’re all locked in there together’, but actually it was the best they’d gone for ages and everything seemed to tick along fine, and actually now as the lockdown is reducing, there’s been a lot more issues…(Willow, daughter to a survivor)*

### Perpetrators Exploiting the Pandemic

Participants were aware that perpetrators were using the pandemic, and the associated restrictions and guidance, to further control and abuse. The most frequent examples people gave were perpetrators capitalising on reduced in-person contact with people outside of a household, and increasing their surveillance and monitoring of all types of communications:

… he’s like an IT expert and she was very scared that he had put some sort of software onto her computer or her email to listen to her or record her, or to track her address or something like that (David, friend to a survivor)

The second group of examples related to the manipulation of child access arrangements to exert control over survivors. Jean’s daughter, for example, was being coerced by her ex-husband into living with him:

*…he was being a bit difficult. He was saying that the children couldn’t come and stay with her during lockdown because of the [situation with her current partner], so he wanted [her] to go and stay with him, at his house (Jean, mother to a victim)*

Additionally, a few people described how the abuse had transformed. Esther, for example, was supporting a friend whose mother was the perpetrator. She had hoped the social restrictions would improve the situation, since her friend lived apart from her mother, however, the perpetrator simply intensified her verbal and emotional abuse:

*I was going to say it eased, it hasn’t eased. On one hand, because by law, they couldn’t meet…so that wasn’t there, but she still has experienced a lot of verbal, psychological, emotional abuse over the telephone…I would say that shows, doesn’t it, the burden of the verbal abuse, and how severe it is, that without the physical contact, how traumatic it continues to be (Esther, friend to a survivor of family abuse)*

Jean also described how the harassment and intimidation of her daughter had escalated during the pandemic, with the perpetrator completely flouting restrictions:

*I’ve been to see neighbors and they said they were seeing him during lockdown, he was intimidating her around the neighborhood, but she never told us (Jean, mother to a victim)*

Another participant, Richard, described how the transfer to online court cases, during the pandemic, had resulted in his friend’s experience of partner abuse being compounded by organisations supposed to ensure justice. The perpetrator had exploited the survivor’s lack of technological know-how, and had stopped her from accessing appropriate equipment and space:

*My friend, who is not a computer bod was really struggling, even with the idea of using a computer. Full stop. So, to go beyond that, to running commercial software, needing high power broadband facilities, which she’s never used before, when trying to defend her actions against an abuser in a court environment, is utterly ridiculous and there’s no other word for it…What’s more she was not in her own home because the husband was there. He refused to budge, he said, ‘I’m doing the court hearing here, you’ve got to go elsewhere’. She had nowhere to go, so she ended up doing the whole court hearing on a park bench (Richard, friend to a survivor)*

In this case, the courts were adamant that Richard’s friend had to undertake the hearings alone, so supporters were unable to even sit with her and, in Richard’s words, the survivor was ‘*driven to a nervous breakdown’.*

In addition to perpetrators using the pandemic to further abuse and control survivors, there were also a few examples given where perpetrators had additionally targeted those trying to offer informal support. Maria, a neighbor of a survivor, felt that she had limited options to escape from the perpetrator’s intimidating behaviour, because of her proximity to the situation:

…*the police have assured me that he would get told that he couldn’t approach me or anything, but in the past week, I guess we are all at home now, three times now he has come over and talked to me. And after three times I have reported it to the police, ‘cos I feel very scared by him…(Maria, neighbor and friend to a survivor)*

### Offering Support Within the Context of a Pandemic

People offering informal support do so within the context and complexity of their own lives, and the pandemic has made everyone’s life different, and potentially more difficult:

*I think it just made everything very much more intense (Gwen, aunt to a survivor)*

Participants in this study described how the effects of the pandemic on their everyday lives had impacted the way they were able to offer support and to cope with the DA situation.

### Protecting Health and Sheltering

One of the primary impacts of the pandemic was action required to protect one’s own health. Many people described not being able to see the woman they were supporting face-to-face for a period of time, due to social restrictions preventing the spread of COVID-19. Furthermore, a couple of participants, mentioned the impossibility of any in-person contact, either because they themselves needed to shield from the virus (due to age or health status), or because they lived with someone else who needed to. Fiona described how her mother had accompanied her sister in the past for the handover of the children to their father. Her mother had to self-isolate during the pandemic, and thus this role had become Fiona’s responsibility. Richard talked about not being able to see his friend, due to his wife being at risk health-wise:

*My wife is actually in a very vulnerable situation. She has [poor health] and is awaiting a major operation and she has been instructed not to go on public transport or to come into contact with anybody who might possibly be carrying the virus and, of course, that applies equally to me (Richard, friend to a survivor)*

### Feeling Isolated

In addition to being less able to see the survivor, participants also described how the limitations of contact with people in their own social network had impacted them. Several people relied on their friends and family to help them cope with supporting a survivor and, with this input much reduced, were finding it harder to manage. In particular, it could leave people feeling isolated, with participants indicating that the possibility of social interaction had been a motivating factor regarding participation in the research:

*I’ve found the whole thing very isolating, so I was hoping that maybe talking to someone might help me (Maria, neighbor and friend to a survivor)*

Where there was more than one person involved in support provision, a couple of participants described making decisions about whether or not it was appropriate to burden others if they lived too far away to easily respond during the pandemic:

*… other members of the family live further away and I’m the person who is closest to her physically, as a family member, but then sometimes it’s been ‘Should I worry other people?’ or, ‘What’s important for them to know, but what’s just going to worry them without them being able to do anything with that?’(Gwen, aunt to a survivor)*

### Capacity to Help

Some participants mentioned how stretched their capacity had been during the pandemic, as they adjusted to working from home, provided additional care for children, and helped with home-schooling. Beyond this were the everyday tasks that just took so much longer:

*… because my wife’s health situation is [poor], I’m the one who has to actually go out do all the shopping...what should normally take perhaps half an hour for shopping ends up taking three or four hours, and that in itself is stressful together with trying to deal with what’s required [to help my friend] (Richard, friend to a survivor)*

Because everything seemed to require so much thinking, planning and extra time, supporting a woman experiencing abuse alongside this really tested people’s resilience. Others indicated that whilst the mode of support had somewhat altered, they felt that reduced options for social activities meant that they had more time available to help:

*In a sense there’s more time to support, because even though we have work and commitments, we’re not going out socialising (Esther, friend to a survivor of family abuse)*

### Unable to Escape

Several participants spoke about survivors being unable to leave the relationship during the pandemic. However, they were not the only people trapped by the social restrictions. Maria, living next door to her friend and the perpetrator, was desperate to move away:

*…the sergeant at last rang me back and he said, ‘Well what do you want me to do?’ and I was like, ‘I am trying to move out of this house’, I’m due to move out of this place next week, but who knows if this is going to happen now…(Maria, neighbor and friend to a survivor)*

Unfortunately, the police response to her predicament was largely unhelpful, and Maria felt that they were failing to appreciate just how stressful and scary it was living 24/7 next to a dangerous, unpredictable, and intimidating man.

### Finding Creative Solutions

As the impact of the pandemic, and the associated social restrictions, became apparent to people, they described thinking creatively about how they could continue offering support. Many expressed decisions that they had made to override or defy the restrictions if it became necessary to do so, in order to protect the survivor:

*The difference was when lockdown was very strict, I always knew in my head that if I needed to go and see her, I would, and I didn’t have any difficulty that I would justify that (Gwen, aunt to a survivor)*

However, in the vast majority of cases, people had acted within the guidance, even if they had not realised this at the time.

#### Support Bubbles with Survivors

Soon after the initial nationwide lockdown in March 2020, the UK government introduced the possibility of creating a support bubble with lone adult households, to help people who were cut off from family and friends. In several situations, where the survivor had left the relationship, or was about to, participants considered whether or not to bubble with her:

*And in lockdown, I saw a lot of her in lockdown. I broke the lockdown rules, because I said, ‘I’m not having you on your own here, you can still come round to the house’. So, we kept our own little bubble going (Jean, mother to a victim)*

…*when she moved was when the policy around bubbling came in, so we said we would bubble with her, and we went down there and stayed with her on the first long weekend after she moved (Laura, friend to a survivor)*

There was a potential cost to this decision, since only one support bubble was permitted, but those who had formed a bubble with a survivor were clear that this was the right choice:

*What we did is, when we knew about the bubbling, we decided to bubble with her, because I don’t have family here, but I have a lot of friends who are single or lonely, and my wife she has her parents here, but we didn’t bubble with them, so we decided to bubble with this friend because she was planning on leaving the house before the divorce…(David, friend to a survivor)*

#### Monitoring the Situation

Given the possibility of increased surveillance by perpetrators of any communications between informal supporters and survivors, several participants indicated alternative ways by which they tried to assess what was happening. Fabian’s regular socialising with his friend had stopped, and knowing that their WhatsApp messages were being monitored, he altered his communications to generic check-in messages, capitalising on anything that was happening in his friend’s life, in order to keep channels of communication open, without alerting the perpetrator.

Teresa had been secretly meeting her friend prior to the pandemic, helping her make plans to leave her husband. The limitations in face-to-face communication, due to the social restrictions, made this impossible, and any other form of contact would have drawn the perpetrator’s attention. Thus, they began communicating via a legitimate contact who was also aware of the situation, the friend’s adult daughter:

*So, we were then in the situation where I wouldn’t be able to have direct contact with her without him knowing, and we can’t trust her phone because we’re pretty sure that he monitors her mobile phone…her daughter is in contact with her, because that would be a kind of acceptable contact, whereas my contact would be a bit odd, really, in terms of what the perpetrator would see. Any information or messaging, or anything I would like to give to her, I do that through [her daughter] (Teresa, friend to a survivor)*

For Willow too, her primary way of monitoring the situation was through another person; receiving daily telephone reports from her sister, who was living with her mother and the perpetrator. Gwen was able to safely contact her niece, but was keen not to overwhelm her with frequent messages, so instead kept an eye on social media and group chats to get a sense of how her niece was doing, and whether or not she was interacting with others:

*… I’d make sure I knew how she was, that she was talking to somebody, it didn’t need to be me, but I was monitoring, so if I saw her posting something on Facebook, or we’ve got a family WhatsApp group, and I’d know what was going on from that sort of thing (Gwen, aunt to a survivor)*

#### Bridging the Gap?

Many participants recognised that specialist and public services might be struggling with capacity, or in their delivery of services by usual means. They were aware that it might be necessary for them, as informal supporters, to bridge the gap between that which was available usually, and that which was currently accessible:

*Anyway, during lockdown, she wasn’t seeing her doctor as regularly, and the IRIS worker*^4^* finished, she closed the case... So, during lockdown, I was seeing a lot of her (Jean, mother to a victim)*

In particular, informal supporters became increasingly aware of the extra challenges for survivors who wanted to leave the relationship, and their home, during the pandemic. Teresa was supporting a friend to exit a relationship at her own pace until the pandemic hit, and described the consequent pause in progress as both ‘challenging’ and ‘uncomfortable’:

*It feels like she’s in a situation where she was making moves to leave…There would be no slow exit, which is what she was looking to do. It would be a bit of a furore to do it in a lockdown situation (Teresa, friend to a survivor)*

David and Laura were able to help a survivor to leave her husband, and described the extreme challenges for their friend in planning and implementing her exit, including creating multiple reasons to leave the house daily in order to phone friends, support services, solicitors, estate agents, banks, sellers of white-goods and furnishings, and house-movers:

*…there were times when she took out her daughter, the moments for making phone calls and stuff, and was phoning the local support service and lawyers and legal aid people, and stuff with her daughter in the car, and trying to distract her and feed her snacks, and stop her from crying, while trying to wrap her brain around all of this stuff. And yeah, it struck me that the logistics were just so hard, because how do you leave when he is there all the time? And how do you pack up your stuff and make all the right arrangements and stuff without him knowing (Laura, friend to a survivor)*

The move was only possible with a team-effort; David and Laura stepped in to provide large amounts of childcare, and practical and emotional support over a very intense period:

*…we have been going to her new place for two long weekends and we stay there for four days, two times, and we helped her to settle, we found furniture to help her. I was mainly taking care of the child…she couldn’t go to a shop to buy stuff, there were some shops that were delivering washing machines and fridges and all that, but it took weeks to arrive. So basically, we were going around with our car trying to find a table, using Facebook marketplace. Getting in contact with people, getting in contact with her, she doesn’t have a Facebook account because she doesn’t want to be tracked by the husband… we were there for three, four days and I was exhausted, it was very, very tiring. Second week we were there for five days and by the end of it I said to my partner, ‘we can’t come back next week because it’s too much’ (David, friend to a survivor)*

David was clear that whilst he had had some flexibility and capacity, many people would not, and that without this, it would have been exceptionally difficult to provide the level of support needed to help someone leave and set up home in the midst of the pandemic.

Moreover, people described occasions where professionals had simply asked too much of them, for example Maria felt unable to say ‘no’ to the police when they asked her to accommodate her neighbor for the second time:

*...she’s stayed in my house now twice, when she should have gone into a refuge, and given, you know, specialist support rather than being in my house, with him being my next-door neighbor (Maria, neighbor and friend to a survivor)*

## Discussion

In their accounts, the friends, relatives, neighbors, and colleagues of DA survivors described the additional challenges presented by the pandemic, both for the women experiencing abuse, and for themselves as they tried to help. On the whole, informal supporters struggled to remain informed about what was happening, but some were aware that the perpetrator was using the pandemic, and the related restrictions, to his advantage; to further control, coerce, abuse, and isolate. Since the pandemic had made informal supporters’ day-to-day lives more complex and demanding, it was from an already-stretched position that people were attempting to offer support. None-the-less, informal supporters remained keen to assist survivors, often finding creative solutions to achieve this.

Previous research has shown that many people are willing to offer support, and to undertake an informal supportive role with a woman experiencing abuse (Beeble et al., 2008). And, this appears to be the case whether the people are relative strangers (Borsky et al., 2018; Gainsbury et al., 2020), or in much closer relationships with a survivor (Banyard et al., 2009; Latta, 2008). What is heartening in the current study, is that people remained motivated to help, even when DA situations (and many aspects of their own lives) had been further complicated by an all-encompassing global pandemic. In fact, people expressed strong levels of commitment and self-sacrifice, including being ready and willing to override any governmental or legal directives which might prevent them from offering support.

These findings give an indication of the endurance of informal support, even in the face of adversity, when life circumstances are especially difficult. This reinforces previous findings about the persistence of care provided by individuals, even when domestic abuse situations are complex, or continue over long periods (Gregory et al., 2019; Latta, 2008). Additionally, our findings demonstrate the ingenuity of informal supporters, with people adapting modes and types of communication, thwarting perpetrator behaviours, and utilising any opportunities the pandemic provided. This parallels the creativity and innovation noted within specialist services during this time (Tierolf et al., 2020). Perhaps necessity, during the current pandemic, really has been ‘the mother of invention’.

Kelly describes the idea of reduced ‘space for action’ in abusive relationships, where thinking and opportunities for agency narrow as the survivor adapts behaviour and attempts to live as the perpetrator demands (Kelly, 2003). From this position, it becomes increasingly difficult to imagine life outside of this control, where there is freedom to act. During the pandemic, this space has further reduced for many survivors, not least in the domains relating to ‘support and relationships’ and ‘wider community’ (Sharp-Jeffs et al., 2018). Within these domains there have also been corresponding limitations, during the pandemic, for informal supporters. The social restrictions imposed to prevent the spread of the virus, combined with perpetrators actions to increase surveillance and monitoring, and control survivors’ interactions, similarly challenge the space for action by informal supporters, in providing the most helpful forms of assistance—emotional and tangible support (Goodkind et al., 2003). Of course, informal supporters are much less under the control of perpetrators, and much less at risk from his behaviours, but the pooled effect is diminished agency (Bagwell-Gray & Bartholmey, 2020).

Our findings support recently published reports highlighting pandemic-specific forms of abuse used by perpetrators (Davidge, 2020; Gingrich, 2020; Newberry & Santa Cruz, 2020). This is dispiriting, but perhaps unsurprising. We know that perpetrators of DA actively and relentlessly look for new ways to exert control over the person they are abusing (Stark, 2007), and unfortunately COVID-19 has presented a plethora of opportunities. The perpetrator behaviour, which many informal supporters mentioned, was increased surveillance and monitoring. This presents a challenge, particularly around survivors accessing online support. On the one hand, there has been a big move towards the provision of online support for survivors by specialist DA services during the pandemic, and there has been substantial uptake of this form of support (Office for National Statistics, 2020). On the other hand, survivors may be unaware of the level of digital surveillance employed, making this mode of communication less safe than they realise. Organisations need to carefully consider whether the extensive move to online services will work for everyone, and to ensure that they actively highlight to survivors the potential dangers of engaging in this way. Beyond perpetrator behaviours towards survivors, there is also an issue for close proximity allies, since social distancing measures and extensive working from home have made it difficult (for neighbors, in particular) to escape direct perpetrator behaviours. Previous research has indicated that informal supporters may become an additional target for perpetrator behaviour (Gregory et al., 2017a; Gregory, 2017c), and thus there is a possibility that secondary victimisation of informal supporters may increase as a result of the pandemic.

Apparent in a few of the narratives, was the potential for increased harm and/or complicity with perpetrators’ abuse by professionals and organisations, particularly those involved in justice processes. Whether through lack of understanding, lack of competence, or with intent, these actions caused survivors, and those informally supporting them, additional harm. Organisational harm and abuse is something which can happen in usual times; almost a decade ago, Hester described the systemic contradictions and organisational cultures which not only disserve DA survivors but, on occasion, support perpetrators (Hester, 2011). However, the organisational harm experienced during the pandemic has been further complicated by ill-thought through changes to processes, which have been difficult for people to challenge.

Regarding the gendered impact of the pandemic, including providing informal support to DA survivors, there were some differences regarding the experiences and toll on the women and men who participated, but by far the biggest difference was the proportion of women and men who stepped forward to take part in this research (5:1). It is possible that this is an indicator of survivors’ preferences for disclosing to another woman, but it may also suggest that women are more likely to discern abuse and to want to offer support. At a time when women are already ‘bearing the emotional brunt’ of COVID-19 (The Fawcett Society, 2020), greater recognition of their societal contribution is warranted. Additionally, cursory recommendations for community initiatives to tackle and respond to DA, need to consider how such schemes can actively engage men, rather than simply re-creating an over-reliance on women’s benevolence.

Furthermore, whilst informal supporters have indeed shown themselves to be both committed and creative during this time, it would be wrong to pursue community initiatives as an easy solution or a quick fix. To do so would be a disservice to survivors, their informal supporters, and to specialist service providers. The impact of providing informal support is significant and, as indicated in our findings here and elsewhere, takes no small amount of capacity, energy, and emotion, within the context of people’s everyday lives (Gregory et al., , 2017b, 2019). It is not an employed, reimbursed, or acknowledged role (unlike other recognised and compensated carer roles), and is often boundless. Rather than advocating for a simplistic reliance on individuals who are heavily invested, but untrained and ill-equipped, or a disinvestment in the professional sector, we need to find more inspired solutions. Initiatives, such as the *Ask Me* community training run by Women’s Aid in the UK, could form part of the solution, though this type of programme is likely to be resource intensive. Further research with informal supporters is needed to ascertain how to best support and equip them without imposing an impossible burden.

### Strengths and Limitations

The primary strength of this research is originality of perspective, which is important for two reasons. By considering situations of DA from less usual vantage points, different facets and different opportunities for intervention became apparent. In particular, understanding how situations of DA appear to those willing to offer support, enables us to work towards better equipping friends, relatives, colleagues, and neighbors to respond well. The second relates to the potential risks of conducting direct research with survivors during the pandemic; by collecting data from those connected with the situation, but not in immediate danger, we gain insights into situations of DA during this period. Additionally, the flexibility and responsiveness of the data collection and analysis has created an opportunity for timely, applicable findings, beneficial in a thus on-going global pandemic.

The limitations of this research are twofold. Data from a relatively small number of people have been included in the secondary analysis, and thus it is conceivable that we have not captured the full range of informal supporter experiences. Moreover, since the findings described here are from a secondary analysis, they may not reflect the complete range of narratives encountered if people had been recruited specifically to explore their experiences of providing support in relation to the pandemic.

## Conclusion

Positive support from friends, family members, neighbors, and colleagues is often critical for women experiencing domestic abuse. The COVID-19 pandemic, and associated social restrictions, have amplified the experiences of survivors, and have presented challenges to people offering help. Whilst direct research with survivors has been curtailed for reasons of safety during the pandemic, it has been possible to gain insights through connected third parties. Informal supporters are sometimes aware of perpetrators’ abuse tactics and operate creatively in their attempts to remain in contact and to continue offering support to survivors. Assisting a survivor in usual times can be demanding and burdensome. Because the pandemic has immensely impacted everyday life, informal supporters are offering help from an already-stretched position. Despite the difficulties faced, many informal supporters remain keen to help. Friends, family members, neighbors, and colleagues of survivors are often deeply enmeshed in DA situations, but frequently lack training and feel out-of-their-depth. Informal support is no replacement for specialist support but is often an important adjunct or gateway to professional help. As such, we need to empower and equip informal supporters to offer help, without imposing an impossible burden on them.

## Footnotes

1 https://www.womensaid.org.uk/

2 https://www.womensaid.org.uk/our-approach-change-that-lasts/askme/

3 Examples include: https://safelives.org.uk/news-views/domestic-abuse-and-covid-19#family-friends and https://www.womensaid.org.uk/wp-content/uploads/2020/05/NEW_For-Community_COVID-19-and-Domestic-Abuse-Sexual-Violence-and-GBV_Galop-addition.pdf

4 An IRIS worker is an advocate based in a local specialist DA service. The advocate works alongside clinicians in general practice, receiving referrals of women experiencing domestic abuse. For further information: https://irisi.org/iris/about-the-iris-programme/
